# Injectable Biodegradable Silica Depot: Two Months of Sustained Release of the Blood Glucose Lowering Peptide, Pramlintide

**DOI:** 10.3390/pharmaceutics14030553

**Published:** 2022-03-02

**Authors:** Puneet Tyagi, Mika Koskinen, Jari Mikkola, Sanjay Sarkhel, Lasse Leino, Asha Seth, Shimona Madalli, Sarah Will, Victor G. Howard, Helen Brant, Dominic Corkill

**Affiliations:** 1 Dosage Form Design and Development, BioPharmaceuticals R&D, AstraZeneca, Gaithersburg, MD 20874, USA; 2 DelSiTech Ltd., PharmaCity, Itäinen Pitkäkatu 4 B, 20520 Turku, Finland; mika.k.koskinen@delsitech.com (M.K.); jari.mikkola@delsitech.com (J.M.); sanjay.sarkhel@uef.fi (S.S.); lasse.leino@delsitech.com (L.L.); 3 Renal BioScience, Early CVRM, BioPharmaceuticals R&D, AstraZeneca, Cambridge CB21 6GP, UK; asha.seth@astrazeneca.com (A.S.); shimonap7@gmail.com (S.M.); 4 Metabolism BioScience, Early CVRM, BioPharmaceuticals R&D, AstraZeneca, Gaithersburg, MD 20878, USA; sarah.will@astrazeneca.com (S.W.); victor.howard@sparktx.com (V.G.H.); 5 Animal Science & Technologies UK, Clinical Pharmacology & Safety Sciences, AstraZeneca, Cambridge CB21 6GP, UK; helen.brant@kymab.com; 6 Early R&I BioPharmaceuticals R&D, AstraZeneca, Cambridge CB21 6GP, UK; dominic.corkill@astrazeneca.com

**Keywords:** long acting, sustained delivery, biologics, silica microparticles

## Abstract

Diabetes mellitus is a major healthcare challenge. Pramlintide, a peptide analogue of the hormone amylin, is currently used as an adjunct with insulin for patients who fail to achieve glycemic control with only insulin therapy. However, hypoglycemia is the dominant risk factor associated with such approaches and careful dosing of both drugs is needed. To mitigate this risk factor and compliance issues related to multiple dosing of different drugs, sustained delivery of Pramlintide from silica depot administered subcutaneously (SC) was investigated in a rat model. The pramlintide-silica microparticle hydrogel depot was formulated by spray drying of silica sol-gels. In vitro dissolution tests revealed an initial burst of pramlintide followed by controlled release due to the dissolution of the silica matrix. At higher dosing, pramlintide released from subcutaneously administered silica depot in rats showed a steady concentration of 500 pM in serum for 60 days. Released pramlintide retained its pharmacological activity in vivo, as evidenced by loss of weight. The biodegradable silica matrix offers a sustained release of pramlintide for at least two months in the rat model and shows potential for clinical applications.

## 1. Introduction

Diabetes mellitus poses a major global health challenge, with estimates of the human population affected to touch a staggering 783 million by 2045 [1]. It results from a complete deficiency of insulin (type 1) or impaired insulin secretion (type II) due to β-cell dysfunction. Insulin and related β-cell hormones regulate postprandial glucose regulation by decreasing glucagon secretion [2,3,4] and slowing gastric emptying. The disease imposes a huge burden on the healthcare system and accounts for more than 10% of its spending [1]. It manifests multiple complications, and an active diabetes care and management program aims at regulating the blood glycemic levels [5]. Therapeutic interventions range from lifestyle changes, drugs, smart delivery technologies, and regenerative medicine [3,5,6,7,8]. Although therapeutic drugs have some limitations, they continue to be the main choice in the active management of diabetes care. Currently, the most widely used therapeutic for diabetes control is based on the endogenous hormone, insulin, and its analogues [7,9]. However, due to their complex molecular structure and physicochemical properties, formulation and delivery of these peptides need due consideration [10,11,12,13,14]. Parenteral routes, such as intravenous, intramuscular, and subcutaneous are the most popular routes of delivery for these peptides.

A major challenge in diabetes management concerns regulating the varying amounts of blood glucose during the course of a day. In response to dietary food intake, glucose metabolism is carried out by the pancreas by secreting insulin and the related hormone amylin. Amylin complements insulin in the regulation of postprandial glucose by decreasing glucagon release, slowing gastric emptying, and decreasing food intake [15]. Therapeutic intervention by insulin and amylin-based peptides to regulate blood glucose metabolism in diabetes is thus a logical mimic of the endogenous system. A synthetic analogue of amylin, pramlintide acetate (Mw. = 3951.41) is used as an adjunct to preprandial insulin therapy in diabetic patients who fail to achieve glycemic control with insulin therapy alone [16,17]. The pharmacokinetic (PK) profile of blood glucose-lowering drugs is key in defining their in vivo efficacy. Blood glucose levels vary within a day depending on the timing and nature of food intake. A drug with an inadequate PK profile will lack efficacy in regulating glucose levels and consequently might need multiple dosing. Excessive moderation of glucose levels might result in hypoglycemic conditions and may prove fatal [18]. Engineering the PK profile of a glucose-lowering drug is an area of intense investigation and has resulted in various analogues of insulin designed to be active from short-acting up to long-acting [7]. In this context, reports from clinical studies seem to suggest that maintaining a threshold level of glucose-lowering hormones may better facilitate the management of diabetes [19,20]. Sustained delivery systems such as implants made from a mixture of insulin and micro-recrystallized palmitic acid (marketed as Linbit™ and Linplant™) have shown better therapeutic efficacy as compared to daily insulin injections in rodents [21]. Although insulin implants show promise, they require surgical intervention. This is not only a burden to the healthcare system but also a concern for patient compliance. Sustained injectable drug delivery systems have shown promise and offer the advantage of avoiding the need for repeated dosing to achieve therapeutic effects. Similar to pramlintide activity, GLP-1 peptide elicits insulinotropic effects by binding to the GLP-1 receptor, thereby regulating insulin and glucagon secretion. However, endogenous GLP-1 is rapidly degraded with a 1.5–5 min half-life [22,23]. Nanoparticle-based drug delivery systems have been often employed to enhance the bioavailability of drugs. For example, Choi et al. [24] prepared a copolymer of poly [(dl-lactic acid-co-glycolic acid)-b-ethylene glycol-b-(dl-lactic acid-co-glycolic acid)] that undergoes a temperature-dependent reversible solution–gel transition for in situ depot formation to deliver GLP-1. However, most polymer-based sustained delivery systems lack clinical translation due to limitations issues related to reproducible scale-up of mono-disperse particles [25,26]. In a very recent report, GLP-1 fused recombinantly to elastin-like polypeptide (ELP), has been shown to elicit zero-order release kinetics from a subcutaneous depot and circulation times (also glycemic control) up to 10 days in mice [27].

In this communication, we highlight a sustained delivery strategy for in vivo delivery of the synthetic peptide analogue of amylin, pramlintide acetate. As mentioned earlier, amylin is the endogenous hormone co-secreted with insulin by the pancreas for glucose metabolism in vivo. Clinical investigations seem to suggest that there is an increased risk of severe hypoglycemia (particularly in type I diabetes) when adjunctive therapy is used with insulin and careful dosing is required for patient safety [18]. Such effects can be moderated either by decreasing the single dosing of preprandial insulin/pramlintide or by a sustained long-term slow release of the hormone(s) [19]. Sustained slow delivery of pramlintide might mitigate the need for separate administration of the two drugs by injection each time before a meal and might improve patient compliance. We present here the formulation and in vivo (rat model) release profile of pramlintide from our proprietary silica-based delivery matrix [28,29,30]. Silica offers numerous advantages as compared to other sustained release systems. It is biodegradable, offers formulation under mild aqueous conditions and its degradation product, silicic acid (weak acid, pKa 9.84) does not acidify the environment and is non-toxic. This presents a significant advantage for formulation (in near-native conditions) and delivery (degraded products do not destabilise protein structure) of biologicals [25,31].

## 2. Materials and Methods

### 2.1. Materials

All analytical grade reagents and solvents, including tetraethyl orthosilicate (TEOS) and standard solution for silicon atomic absorption, were purchased from Sigma-Aldrich (St. Louis, MO, USA). Pramlintide acetate was obtained from MedImmune, Inc. (Gaithersburg, MD, USA).

### 2.2. Preparation of Silica Microparticles

TEOS was hydrolyzed in the presence of deionized water and 0.1 M HCl to prepare the silica sol. TEOS/water/HCl at a molar ratio of 1:15:0.005 was hydrolyzed at room temperature, under vigorous stirring for 25 min. Following hydrolysis, silica sol was stored in an ice bath for 1 h, and the pH adjusted to 2.6. Aqueous solution of pramlintide (10 mg/mL) was mixed with the silica sol to yield 5% *w*/*w* pramlintide load while stirring vigorously. The silica sol–pramlintide mixture was further diluted with water to attain a molar ratio of TEOS/water to 1:150 at pH 4.0. A mini spray dryer (Büchi B-191, Flawil, Switzerland) was used to spray dry the silica sol–pramlintide having inlet and outlet temperatures of 125 °C and 60 °C, respectively, at the feed rate of 4 mL/min.

### 2.3. Preparation of Pramlintide-Silica Microparticle Silica Hydrogel Depot Formulation

Pramlintide-silica depot was prepared by combining the spray-dried pramlintide-silica microparticles with R400 silica sol. As mentioned earlier, the R400 silica sol was prepared by acid-catalysed (pH 2.0) hydrolysis of TEOS in a molar ratio (TEOS/water) of 1:400. The R400 sol was added to pramlintide-silica microparticles (1 mL/0.5 g) and magnetically stirred to a suspension. The pH of the suspension was adjusted to 6.3 by dropwise addition of 1 M NaOH. Additionally, 1 mL plastic syringes (Becton-Dickinson, Franklin Lake, NJ, USA # 309628) were filled with the homogenous suspension and capped (Sigma-Aldrich, St. Louis, MO, USA, Monoject syringe tip caps). The suspension was allowed to gel at ambient temperature while maintaining homogeneity by placing the syringes on a rotor (Stuart Rotator SB3).

### 2.4. In Vitro Dissolution Test

To quantify silica dissolution rate, 20 mg of spray-dried pramlintide-silica microparticles were dispersed in 50 mL of 50 mM TRIS buffer (pH 7.4). The microparticle dispersion was kept in a shaking water bath maintained at 37 °C. To quantify pramlintide dissolution rate, pH 7.4 PBS containing 0.01% Tween 80 was used as the dispersing media. Samples were extracted every day and replaced with fresh buffer. Silicon molybdenum blue complex [32] spectrophotometry assay was used to quantify soluble silica. HPLC was used to quantify pramlintide released from the silica depot (described below).

### 2.5. HPLC Analysis of PRAMLINTIDE

The Agilent Technologies 1260 HPLC system with a Waters XBridge protein BEH C4 3.5 µm, 4.6 × 20 mm column was used to measure pramlintide by a reversed-phase HPLC. Mobile phase A included water and trifluoroacetic acid (1000:1, *v*/*v*) and mobile phase B included acetonitrile and trifluoroacetic acid (1000:0.9 *v*/*v*). Mobile phase gradient was 20% B to 65% B over 3 min followed by a reverse gradient from 65% B to 20% B over 30 s and 2.5 min balancing at the end. A 100 µL sample was injected at a flow rate of 1 mL/min, with a column oven temperature of 80 °C. Absorbance was measured at 204 nm.

### 2.6. Total Silica Content Measurement

Dissolved silica was estimated as a soluble silicon molybdenum blue complex. For this, 20–30 mg of pramlintide-silica depot and 10–15 mg of pramlintide microparticles (in a separate experiment) were completely dissolved in 50 mL of 0.5 M NaOH for 48 h at 37 °C. The relative amount of microparticles in the depot was estimated.

### 2.7. Total Pramlintide Content Measurement

The total content of pramlintide within silica microparticles was estimated by organic elemental analysis (OEA) using a FLASH2000 elemental analyzer instrument (Thermo Fisher Scientific, Waltham, NJ, USA) with a CHNS configuration. Analysis was carried out using 1–2 mg of pramlintide-silica microparticles. By measuring the nitrogen content of the sample (OEA method), the amount of pramlintide within the microparticles could be determined as it is the only source of nitrogen within the sample.

### 2.8. Characterization of Pramlintide-silica Microparticles and Depot Formulation

Scanning electron microscopy (SEM) and particle size distribution (PSD) methods were utilised to characterise the pramlintide-silica microparticles. For SEM analysis, LEO Gemini 153 with a Thermo Scientific UltraDry Silicon Drift Detector (SDD) was used, whereas PSD measurements were carried out with a Sympatec HELOS 2370 laser diffraction instrument. Rheological measurements were made using a single rotational rheometer equipped with a parallel-plate with a HPP20 TC measuring geometry (D = 20 mm). Dynamic viscosity was measured by applying shearing force (0.01 to 1000 1/s at 25 °C). Becton Dickinson pre-filled syringes with a 25-G needle (Fine-Ject, Henke Sass Wolf, Tuttlingen, Germany) were used for injection pressure and force measurements. For this, the syringe was placed in a custom-made fixture device with the needle pointing downwards. The load cell of the compression tester was lowered to the point so that it barely touched the plunger (preload less than 0.2 N) and this was recorded as the zero-point. The plunger was compressed with the load cell at a rate of 60 mm/min. Pressure was calculated as a function of the machine extension and the resulting force (N) was estimated by considering the plunger area (16.04 mm^2^) of the Becton Dickinson syringe. NEGYXEN Plus software (Ametek, Largo, FL, USA) was utilised for the measurements.

### 2.9. Endotoxin Analysis

Endotoxin analysis was carried out based on methods outlined in EP 2.6.14/USP<85>.

### 2.10. Pharmacokinetic and Pharmacodynamic Study in Rat

The pharmacokinetic profile was determined in a multidose study in male SD rats weighing approximately 260–290 g. All experiments were conducted at Medimmune, Cambridge, UK, in accordance with the Animals Scientific Procedures Act 1986. Following an acclimatisation period, male SD rats (Charles River, Margate, Kent UK) were assigned to one of two groups, so that bodyweight was equally distributed between the two groups (average weight per group 275 g). Rats were given a single subcutaneous dose of pramlintide microparticle-silica hydrogel depot at a dose of either 5 mg/kg or 15 mg/kg of pramlintide. Pramlintide-silica depot formulation was supplied as a pre-filled syringe, ready to dose. The volume injected was adjusted for each animal to achieve the appropriate mg/kg dose. Serial serum samples obtained from the tail vein were collected from each animal at 6, 24, and 72 h following drug administration. Following this, serum samples were collected at weekly intervals, commencing at one week following dose administration and concluding at eight weeks post-dose. The serum concentration of pramlintide was determined using a human amylin ELISA (Millipore, Darmstadt, Germany EZHA-52K). Graphs and analyses were generated using GraphPad Prism 6.0 (GraphPad Software Inc., La Jolla, CA, USA).

Bodyweight changes were studied in diet-induced obese rats. This experiment was run at AstraZeneca, Gaithersburg, MD, USA according to Institutional Animal Care and Use Committee (IACUC) guidelines. Sprague–Dawley rats arrived at 200–250 g, (Envigo; Indianapolis, IN, USA) were pair-housed and following acclimation, placed on a condensed milk high-fat diet (D12266B, Research Diets; Brunswick, NJ, USA). Following approximately 10 weeks on a high-fat diet, rats were single housed for 2 weeks prior to study start. Rats were sorted into study groups on body weight (average weight per group 444 g). Rats (*n* = 8/group) received a single SC injection of pramlintide microparticle-silica hydrogel at a dose of 3.6, 11, or 22 mg/kg. Vehicle rats received an empty matrix SC injection. Bodyweight was monitored over 28 days.

## 3. Results and Discussion

### 3.1. Preparation of Pramlintide Loaded Silica Microparticles

Acid-catalysed hydrolysis of TEOS resulted in silica sol. The addition of pramlintide solution into the sol encapsulated the peptide within the networked structure of silica. Spray drying yielded pramlintide-loaded non-porous silica microparticles [28,33]. The microparticles were mixed with silica hydrogel (R400) to produce an injectable depot. A load of 3.7% (*w*/*w*) of pramlintide with respect to silica was achieved in microparticle formulation (estimated by OEA analysis). Based on the silica content analysis of the depot, the amount of pramlintide in the depot formulation was estimated to be 11 mg/mL.

### 3.2. SEM Analysis and Particle Size Distribution of the Microparticles

**Figure 1:**
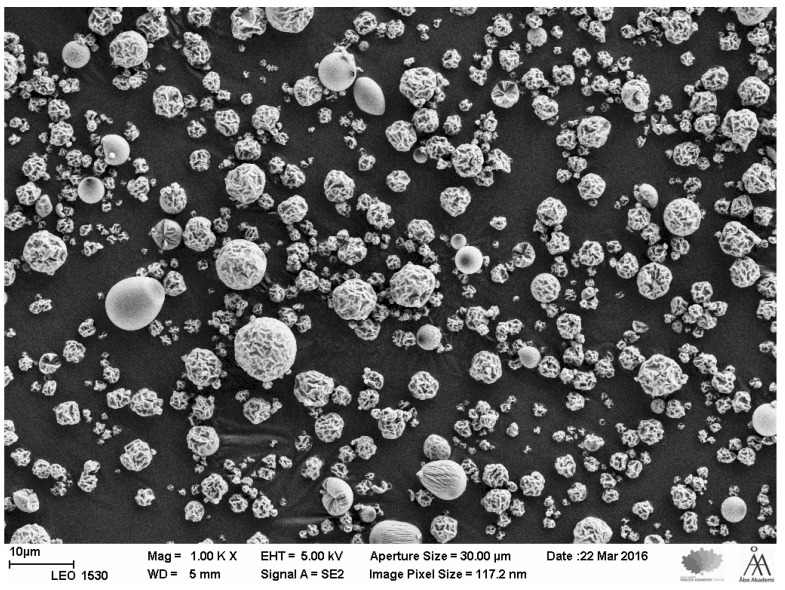
SEM images of pramlintide loaded silica microparticles. Magnification ×1000.

A representative SEM image of pramlintide containing microparticles is shown in Figure 1. The SEM images seem to suggest that particle agglomerates are minimal in the microparticle formulation process. Microparticle shapes tend to vary; this could be due to the spray drying process. PSD measurements (mean ± SD, *n* = 3) were 1.64 ± 0.03 µm, 3.39 ± 0.03 µm, 6.97 ± 0.01, for D10, D50 and D90, respectively. The D value is the diameter at which that % of the sample’s mass is comprised of particles with a diameter less than this value.

### 3.3. Rheology of the Depot Formulation

**Figure 2:**
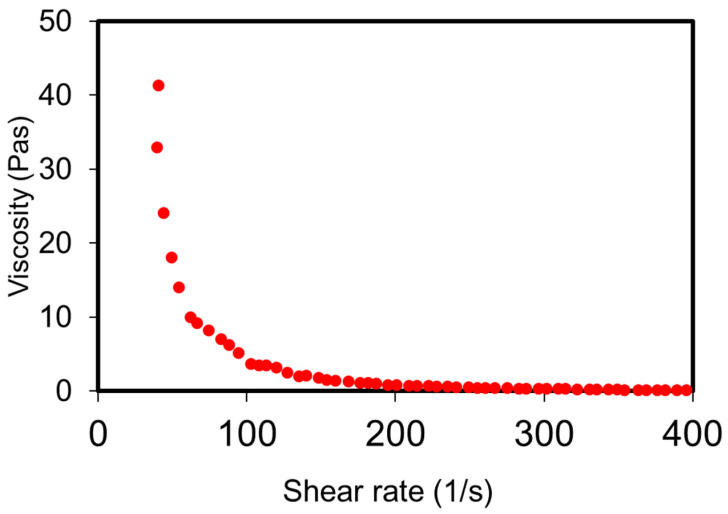
Dynamic viscosity of the pramlintide depot gel formulation in shear rates 0.1 1/s–400 1/s. The data confirm the shear thinning properties of the formulation.

Silica hydrogel, R400 (ca. 60 times the silica saturation levels) stabilises the silica microparticles within the aqueous depot and mitigates issues of sedimentation usually associated with particulate-based delivery systems. Rheology measurements (viscosity vs. shear rate) show that the pramlintide-silica depot formulation has shear thinning properties (Figure 2). This helps maintain the homogeneity of the formulation during storage and, importantly, offers a minimally invasive delivery system by reducing injection force and the use of a small-gauge needle [34].

### 3.4. Injectability of Depot Formulation

**Figure 3:**
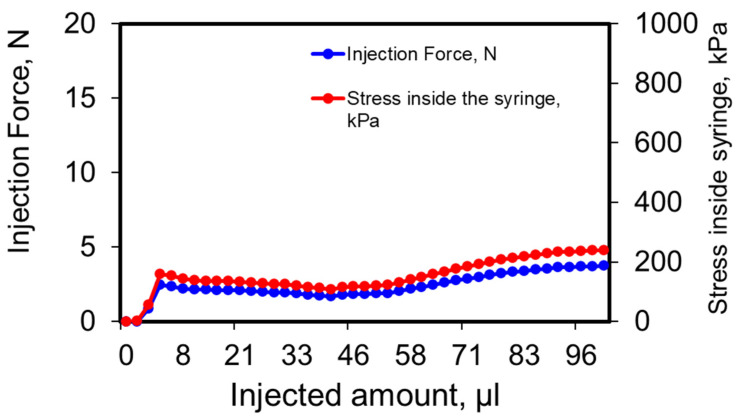
Injection Force and pressure on syringes filled with pramlintide-silica sol-gel system. A 25 G needle was attached to the plunger, and the plunger was pushed at 60 mm/minute.

Injection pressure and force results (Figure 3) show that 100 µL depot formulation could be easily injected with 2–5 N using a 25-G needle. Thus, no significant injection issues are anticipated. On average, the pinch strength of a human hand is about 50 N [35]. The small gauge and the low injection force required to provide a significant advantage as the depot injections are expected to be associated with less pain and incidence of bleeding [36,37].

### 3.5. In Vitro Dissolution

**Figure 4:**
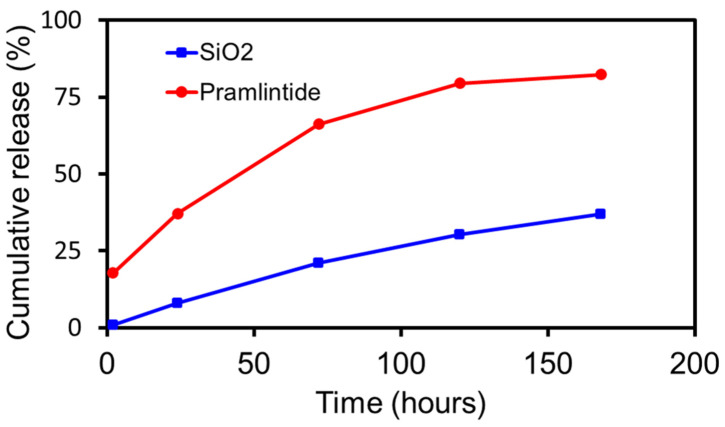
Cumulative estimates of in vitro silica degradation and release of pramlintide from pramlintide-silica depot. Silica dissolution was carried out in 50 mM TRIS–buffer (pH 7.4) and pramlintide release was carried out in PBS (pH 7.4) containing 0.01% Tween 80. Both the studies were carried out at 37 °C under in sink conditions. Data represented as mean for *n* = 3. SD of each data point was less than 2.5%.

Results from in vitro dissolution of pramlintide-silica depot under in sink conditions are depicted in Figure 4. Based on an in vitro–in vivo correlation (IVIVC) factor of 10 for subcutaneous administration [29] and linear progression analysis of the release profile from dissolution studies, it is estimated that pramlintide will have an in vivo release profile of approximately 3–3.5 months. The release of pramlintide from the depot was primarily controlled by matrix degradation at later stages. The initial burst release (ca. 18%) seen could be due to the presence of the peptide near the surface of the microparticle.

### 3.6. Pharmacokinetic and Pharmcodynamic Study in Rat

**Figure 5:**
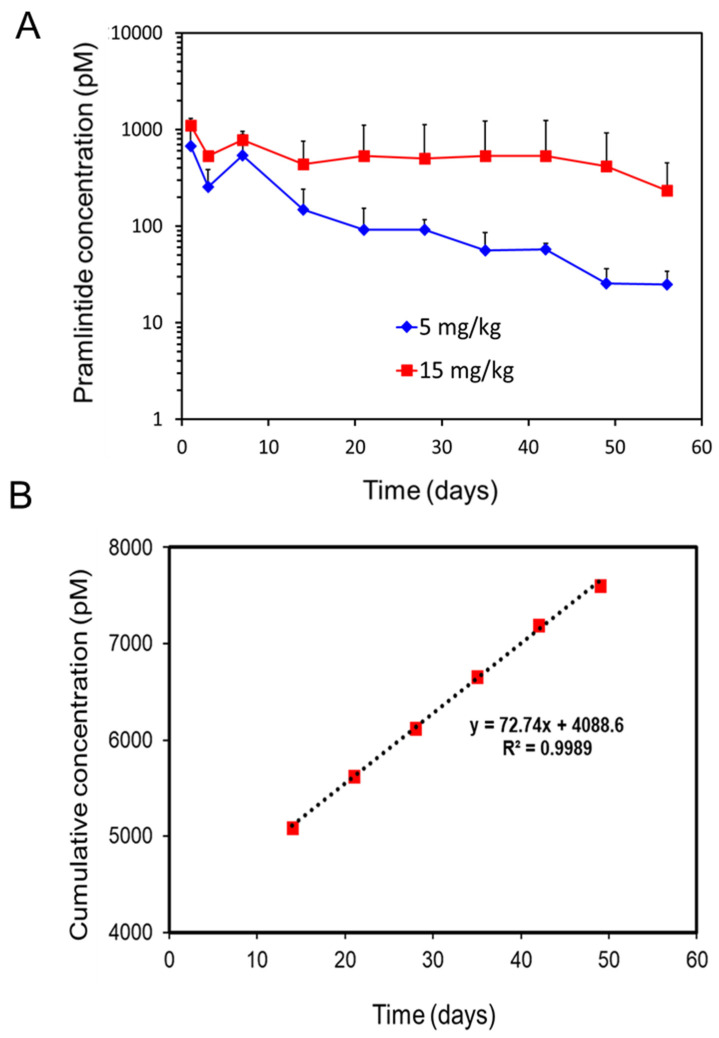
(**A**) Pramlintide plasma concentration in the rat after subcutaneous injection of pramlintide-silica depot at two doses (containing 5 mg/kg and 15 mg/kg pramlintide). Mean and SD, *n* = 5 rats. (**B**) Cumulative concentration vs. time plot depicting the zero-order release of pramlintide from silica depot at higher dosing (15 mg/kg) from 14 to 49 days.

After an assessment of endotoxin levels (<0.06 EU/mg of the depot) within the formulation, an in vivo PK study was conducted in rats. The PK profiles of subcutaneously administered pramlintide-silica depot at two dose levels under a single-dose regimen are shown in Figure 5A. With the higher dosing (15 mg/kg), the plasma concentration of pramlintide stayed over 500 pM for close to two months. After the initial burst, pramlintide release from the silica matrix followed zero-order kinetics (Figure 5B) beyond day 10. A steady concentration of the drug could thus be maintained in the plasma for two months. Extended-release profiles have a potential concern for dose dumping. However, drug release from a matrix, such as silica microparticles, depends on the solubility of the drug in the matrix, as well as the release of an encapsulated drug from microparticles is limited to the rate of silica degradation, which occurs from the exterior of the microparticle inward.

Surprisingly, with the lower dose (5 mg/kg), the serum concentrations showed first-order release as the release dropped from about 500 pM (Day 7) to 20 pM levels by the end of the study (Day 56). The lower pramlintide concentration in the silica might be below the concentration needed to represent an “infinite” reservoir and thus shows first-order release, while the higher pramlintide concentration seems to fulfil the requirement and demonstrates zero-order release (Figure 5B) [38].

In an earlier preclinical study of pramlintide in rats [39], the peak plasma concentration of pramlintide at 50% maximally effective gastric inhibitory dose was reported to be 15 pM. The lower dose (5 mg/kg) formulation in the present study seems to achieve this concentration level by the end of the study.

The results from our study present an interesting comparison with a very recent report of glycemic control by a recombinant construct of GLP-1 fused to thermosensitive ELP [27]. For a single SC injection of 700 nmol/kg of the GLP-1–ELP construct in mice (ca. equivalent of 2.9 mg/kg of GLP-1 peptide), the investigators found that glycemic control (and circulation time) could be achieved for 10 days. In contrast, the silica-based depot system could easily achieve maintaining serum concentration(s) of pramlintide above the therapeutic level (20 pM) for 60 days at both low and high dosing. This observation points out the efficacy of the silica matrix and thus, its potential towards development for sustained release of anti-glycemic agents for clinical applications.

**Figure 6:**
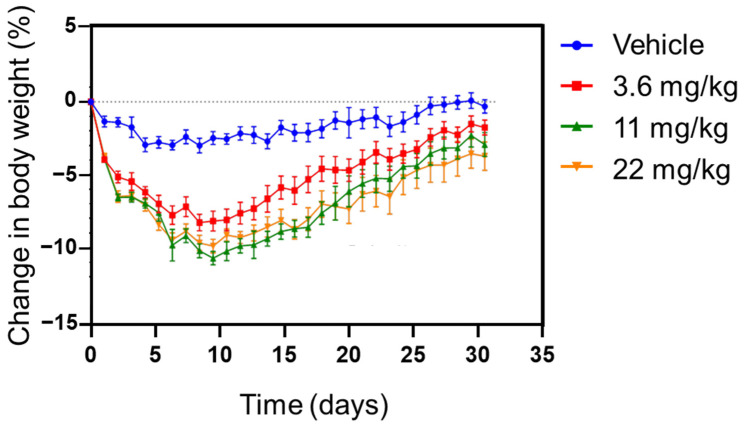
Change in body weight (normalised to % change in body weight in vehicle group) of dietinduced obese rats after a single injection of pramlintide depot containing three different dose strengths. Data is represented as Mean and SEM, *n* = 8 rats.

In agreement with the known pharmacological effect of amylin and its analogues [40], pramlintide depot had a more robust decrease in body weight until about day 9 and then continued to increase throughout the duration of the 29-day study. (Figure 6). This suggests that pramlintide maintains its in vivo activity after release from silica depot. The findings for bodyweight loss mirror very well previously published data in lean rats [41,42,43]. Roth et al. [41] show body weight returning to baseline levels 8 days after the start of amylin treatment in an experiment assessing the effect of continuous subcutaneous amylin infusion over a period of 24 days. Arnelo et al. [42] also show a dose-dependent reduction in food intake and body weight following subcutaneous infusion of synthetic islet amyloid polypeptide by osmotic minipump over an 8-day period in lean rats. At the lowest dose, the effect on food intake was lost after 5 days and after 8 days, the anorectic effect at the top dose was diminishing towards control levels. In another report, significant effects on food intake were seen for the first 6 days when lean animals were administered amylin by minipump over 14 days. After 6 days, food intake returned to control levels despite the continued administration of the drug [43]. Thus, the time course observed for the weight-reducing actions of the pramlintide depot is consistent with the known pharmacology of amylin. The effect of the depot on food intake and body weight in obese rats has also been observed up to 8 weeks [44]. It would also be worthwhile to point out the complexity of dosing and thus, challenges for delivery systems. In an earlier study, at higher doses of pramlintide administered either subcutaneously or intravenously, plasma glucose levels increased. Such glycemic effects were also observed with the administration of amylin in rats [45]. It appears that the glycemic effects of pramlintide and amylin agonists are more pronounced in the fasting state and the glucose-lowering effects are more pronounced in the postprandial state. The effects seem to be more prominent in rodents and of less significance in humans.

## 4. Conclusions

The present study shows the utility of a silica-based matrix for sustained control release of pramlintide in a rat model for two months. The results show that sustained release of pramlintide from silica depot can be achieved to maintain plasma concentration levels of 500 pM. However, optimization of the microparticle formulation is needed to reduce burst release. Further studies are desired to investigate the dosing requirements for achieving therapeutic levels of pramlintide in the serum. This could potentially be achieved by adjusting the drug loading and controlling the rate of silica dissolution. Furthermore, a direct readout of the plasma glucose levels will provide a better estimate of efficacy for the silica-based delivery system. An injectable, scalable, and biodegradable silica-based delivery system has great potential for therapeutic applications in healthcare.

## Data Availability

Not applicable.
